# Quantitative Studies on the Interaction between Saposin-like Proteins and Synthetic Lipid Membranes

**DOI:** 10.3390/mps5010019

**Published:** 2022-02-16

**Authors:** Suzanne I. Sandin, Eva de Alba

**Affiliations:** 1 Department of Bioengineering, School of Engineering, University of California Merced, Merced, CA 95343, USA; ssandin@ucmerced.edu; 2 Chemistry and Biochemistry Ph.D. Program, University of California Merced, Merced, CA 95343, USA

**Keywords:** saposin C, saposin-like proteins, protein-membrane interactions, liposomes, membrane fusion, NMR, dynamic light scattering

## Abstract

Members of the saposin-fold protein family and related proteins sharing a similar fold (saposin-like proteins; SAPLIP) are peripheral-membrane binding proteins that perform essential cellular functions. Saposins and SAPLIPs are abundant in both plant and animal kingdoms, and peripherally bind to lipid membranes to play important roles in lipid transfer and hydrolysis, defense mechanisms, surfactant stabilization, and cell proliferation. However, quantitative studies on the interaction between proteins and membranes are challenging due to the different nature of the two components in relation to size, structure, chemical composition, and polarity. Using liposomes and the saposin-fold member saposin C (sapC) as model systems, we describe here a method to apply solution NMR and dynamic light scattering to study the interaction between SAPLIPs and synthetic membranes at the quantitative level. Specifically, we prove with NMR that sapC binds reversibly to the synthetic membrane in a pH-controlled manner and show the dynamic nature of its fusogenic properties with dynamic light scattering. The method can be used to infer the optimal pH for membrane binding and to determine an apparent dissociation constant (K_Dapp_) for protein-liposome interaction. We propose that these experiments can be applied to other proteins sharing the saposin fold.

## 1. Introduction

Saposins, also known as SAPs (sphingolipid activator proteins), are lysosomal proteins that activate enzymes involved in lipid degradation [1]. Saposins exist in soluble and membrane-bound forms; the latter resulting from peripheral membrane binding [2]. Saposins give name to the saposin fold characterized by four to five alpha helices, six conserved cystines, and a V-shaped open membrane-bound form [3]. The saposin fold is shared by other proteins known as saposin-like proteins (SAPLIPs) displaying a variety of functions that require peripheral membrane binding [4]. SAPLIPs have the capability to dimerize in the presence of micelles and liposomes [3] and some have been shown to induce liposome fusion [5,6]. SAPLIPs exhibit diverse amino acid sequences and are found in different eukaryotic systems performing functions that include sphingolipid catabolism, surfactant stabilization, and antimicrobial activity [4,5].

SAPLIPs have relevant scientific and medical applications beyond their native biological functions. For example, human saposins are used to form lipid nanoparticles [7] that are currently utilized to stabilize transmembrane proteins for high-resolution three-dimensional structural studies [8,9]. NK-lysin is another member of the saposin fold with cytotoxic activity; peptides derived from NK-lysin’s amino acid sequence show antiparasitic properties against *P. dicentrarchi via* membrane disruption of the parasitic protozoa [10]. Surfactant protein B (SP-B), which facilitates respiration in the lungs by reducing the surface tension at the air–liquid interface, also shares the saposin fold [11,12]. However, SP-B has been shown to transfer and exchange lipids but does not permanently interact with membranes [12]. Furthermore, proteoliposomes containing human saposin C (sapC) induce cytotoxicity in glioblastoma cancer cells and are currently undergoing clinical trial [13]. Our lab has recently engineered by recombinant technologies a protein conjugate of sapC with the active domain (BH3) of the pro-apoptotic protein PUMA from the Bcl-2 family [6]. The purpose of this protein chimera (sapC-PUMA) is to retain sapC functionality and simultaneously improve cytotoxicity in cancer cells *via* apoptosis [6]. Our lab has previously reported by NMR that BH3 domains of Bcl-2 proteins adopt significant population of the α-helical conformation in solution and can bind their intended protein targets [14,15,16].

**Figure 1:**
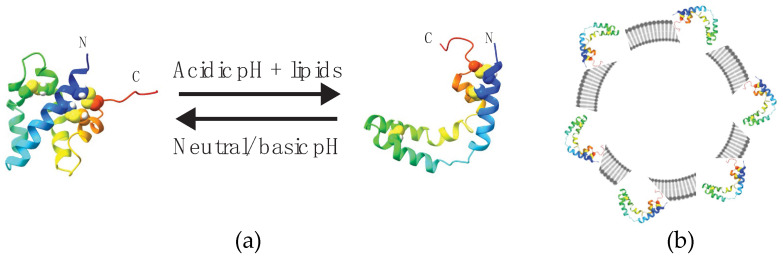
SapC undergoes a pH-dependent conformational change upon peripheral binding to liposomes. (**a**) Ribbon diagrams of soluble (closed form, left) and membrane-bound (V-shape form, right) sapC conformations determined by high-resolution NMR [2,3]. Arrows indicate reversibility with pH in binding and conformational change. (**b**) SapC in open conformation binds to the liposome surface.

We have studied by solution state NMR the binding of sapC and sapC chimeras to liposomes and detergent micelles [2,3,6]. We have been able to determine conformational changes and binding affinity by altering the pH or by increasing the liposome concentration [2,3,6]. High-resolution NMR techniques were originally used to determine the three-dimensional structure of human saposin C in solution and bound to micelles [2,3], representing the protein conformational change from the closed-soluble form to the open micelle-bound form (Figure 1). In addition, NMR studies on the binding of saposin C to liposomes at 1:1 protein:lipid ratio revealed an optimal pH~4 for liposome binding and a membrane-binding pKa of 5.3 [2] (Figure 1). Recently, we have found that sapC-liposome interactions can be maximized by increasing the liposome concentration under mildly acidic conditions (pH 6) [6].

Protein–lipid interactions are traditionally difficult to study by biophysical techniques. For high-resolution NMR, limitations result from particle size due to signal broadening. Large particles, such as liposomes, tumble slowly in solution thus reducing the NMR signal-to-noise ratio to the point where the NMR signals are effectively ‘invisible’ [2,6]. Water-soluble, peripheral membrane binding proteins can be studied by NMR in the unbound conformation. Once the protein binds to liposomes, the resulting proteoliposomes tumble at a rate characteristic of the liposome size, thus turning the protein ‘invisible’ by NMR.

It is reasonable to question whether proteins that bind to liposomes and are capable of lateral movement within the lipid bilayer will show rotational correlation times close to those of the proteins free in solution. To facilitate understanding this concept, the work by Wand et al. on protein NMR in reverse micelles could serve as an ideal example [17]. Extensive evidence from this work shows that the rotational correlation time of proteins tumbling freely in aqueous solutions enclosed by reverse micelles significantly decreases due to the very fast tumbling rate of the reverse micelle [17]. Thus, proteins will show an overall tumbling rate close to that of the particle to which they bind or within which they are enclosed, even if they still undergo translational or rotational movement. Therefore, when considering differences in size such as those described here for SAPLIPs and liposomes, proteins that bind to liposomes will show an overall tumbling rate close to that of the liposome, even if the proteins are freely moving in the lipid bilayer.

It follows that in the presence of an equilibrium between unbound and liposome-bound protein, only the former will contribute to the NMR signal intensity. If the equilibrium shifts toward the bound form, the NMR signal intensity will decrease as the fraction of free protein decreases. This effect in solution NMR spectroscopy can be leveraged to perform quantitative studies on the binding of proteins to liposomes or membranes. Here, we describe the application of this method using sapC–liposome binding as a model that can analogously be applied to other SAPLIPs. Furthermore, we show that the method is applicable to identify changes in liposome binding affinity of sapC mutants designed to improve membrane interactions.

Finally, we describe a method using dynamic light scattering in real time to test the fusogenic capabilities of sapC and several mutants, and to determine the time required for liposome fusion.

## 2. Experimental Design

The main experimental stages of this method include protein expression and purification, production of liposomes, preparation of NMR samples, NMR fast-acquisition experiments for the titration of protein solutions at micromolar concentration with liposomes, and preparation of samples for fusogenic experiments using dynamic light scattering.

**Figure 2:**
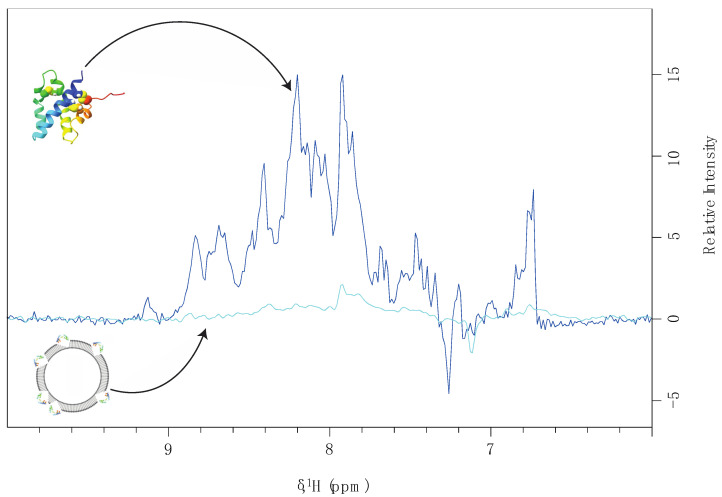
Schematic representation of the effect of liposome binding on NMR signal intensity. NMR signal intensity from soluble sapC in the absence of liposomes (dark blue) and residual intensity in the presence of liposomes resulting from ~5% sapC free in solution (light blue).

A schematic representation of the connection between NMR signal intensity and protein–liposome binding is represented in Figure 2.

### 2.1. Materials

#### 2.1.1. Plasmids for Protein Expression in *E. coli*

- Plasmids for the following constructs were obtained from Gene Universal in the pET-30b vector with a C-terminal six-Histidine tag following a thrombin cleavage site. The following constructs were used in these studies: sapC, sapC-PUMA and sapC-PUMA-DM [6]. The latter construct is a double-mutant (DM) of sapC-PUMA for enhanced protein–liposome binding [6].

#### 2.1.2. Expression Materials

- One Shot BL21 (DE3) Chemically Competent *E. coli* kit (Invitrogen, Waltham, MA, USA, Cat. no.: C600003).
- LB Broth (Fisher Scientific, Waltham, MA, USA, Cat. no.: BP9723-2).
- LB Agar, Lennox (Fisher Scientific, Waltham, MA, USA, Cat. no.: BP9745500).
- Kanamycin sulfate (Thermo Scientific, Waltham, MA, USA, Cat. no.: AC450810100).
- Glycerol (Certified ACS) (Fisher Scientific, Waltham, MA, USA, Cat. no.: G33-4).
- Isopropyl-β-D-thiogalactopyranoside (IPTG) (Fisher Scientific, Waltham, MA, USA, Cat. no.: BP1755100).
- 4–20% Mini-PROTEAN TGX Precast Gel (BioRad, Hercules, CA, USA, Cat. no.: 4561093).
- 4(x) Laemmli Sample Buffer (BioRad, Hercules, CA, USA, Cat. no.: 1610747).
- 2-mercaptoethanol (BioRad, Hercules, CA, USA, Cat. no.: 1610710).

#### 2.1.3. ^15^N Minimal Media Recipe (1 L)

- 867 mL of autoclaved water.
- 100 mL M9 salts pH 7.1: 3 g of KH_2_PO_4_ (J.T. Baker, Cat. no.: 4008-01), 6.8 g of Na_2_HPO_4_ (Fisher Scientific, Waltham, MA, USA, Cat. no.: S374-1), 0.5 g of NaCl (Fisher Scientific, Waltham, MA, USA, Cat. no.: BP358-212). pH solution to 7.1 and filter through 0.45 µm pore.
- 4 g of glucose (VWR, Cat. no.: 0188-1kg) dissolved in 20 mL. Substitute for ^13^C glucose when ^13^C labelling is required.
- 10 mL of 1 g ^15^NH_4_Cl (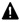 CRITICAL STEP: ensures this is the only nitrogen source).
- 2 mL of 1 M MgSO_4_ (Fisher Scientific, Waltham, MA, USA, Cat. no.: M63-500).
- 1 mL of 1 M CaCl_2_ (Fisher Scientific, Waltham, MA, USA, Cat. no.: C614-500).
- 10 mL of 100(x) MEM vitamin (Gibco, Waltham, MA, USA, Waltham, MA, USA, Cat. no.: 111200052).

#### 2.1.4. Purification Buffers and Reagents

- Tris(hydroxymethyl)aminomethane (Fisher Scientific, Waltham, MA, USA, Cat. no.: T395-1).
- Imidazole (Fisher Scientific, Waltham, MA, USA, Cat. no.: 03196-500).
- HPLC grade water (Fisher Scientific, Waltham, MA, USA, Cat. no.: W5-4).
- HPLC grade acetonitrile (Fisher Scientific, Waltham, MA, USA, Cat. no.: A998-4).
- Nickel Column Buffer A: 20 mM Tris (pH 8.0), 20 mM imidazole, 500 mM NaCl.
- Nickel Column Buffer B: 20 mM Tris (pH 8.0), 500 mM imidazole, 500 mM NaCl.
- Dialysis buffer: 20 mM Tris (pH 8.0), 150 mM NaCl, 2.5 mM CaCl_2_.
- Reverse Phase Buffer A: 95% HPLC grade water, 5% HPLC grade acetonitrile, 0.1% trifluoracetic acid.
- Reverse Phase Buffer B: 95% HPLC grade acetonitrile, 5% HPLC grade water, 0.1% trifluoracetic acid.
- Phenylmethylsulfonyl Fluoride (PMSF) (Thermo Scientific, Waltham, MA, USA, Cat. no.: 36978). Protease Inhibitor: 10 mg of PMSF in 1 mL of isopropanol.
- Cytiva HisTrap FF column (Cytiva, Marlborough, MA, USA, Cat. no.: 17525501).
- Bovine Thrombin (BioPharm Laboratories, LLC, Bluffdale, UT, USA, Cat. no.: 91-030-006). Thrombin (Bovine thrombin) is purchased lyophilized with 6500 units and dissolved with 1.4 mL of dialysis buffer. The thrombin suspension is stored in aliquots of 200 µL at −80 °C.

#### 2.1.5. Lipids

- L-α-phosphatidylserine (Brain, Porcine) (Avanti Polar Lipids, Alabaster, AL, USA, Cat. no.: 840032C-25mg).
- L-α-phosphatidylcholine (Egg, Chicken) (Avanti Polar Lipids, Alabaster, AL, USA, Cat. no.: 840051C-25mg).

### 2.2. Equipment

- Cary 60 UV-vis spectrophotometer (Agilent Technologies, Santa Clara, CA, USA).
- Sorvall Lynx 4000 centrifuge (Thermo Scientific, Waltham, MA, USA, Cat. no.: 75-006-580) with a Fiberlite F12-6x500 LEX rotor (Thermo Scientific, Waltham, MA, USA, Cat. no.: 09-606-2375) installed. Typically, this centrifuge is used for larger volumes at slower speed.
- Branson Ultrasonics SFX150 Sonifier.
- Sorvall wX+ Ultra Series centrifuge (Thermo Scientific, Waltham, MA, USA,) with a Fiberlite F50L-8x39 rotor (Thermo Scientific, Waltham, MA, USA, Cat. no.: 09-608-7051). This ultracentrifuge is used during cell lysis to pellet the cell debris from the supernatant.
- UltiMate™ 3000 Basic Manual System (Thermo Scientific, Waltham, MA, USA).
- C4 reverse phase column (Higgins Analytical).
- Lyophilizer Freezone 2.5 L with a chemistry hybrid pump (LabConco, Kansas City, MO, USA).
- Vortexer (Fisher Scientific, Waltham, MA, USA, Cat. no.: 02-215-41).
- Ultrasonic bath (Fisher Scientific, Waltham, MA, USA, model 15337402).
- Zetasizer Pro Blue Label (Malvern Panalytical, Malvern, United Kingdom).
- Shigemi tube (Sp Wilmad-LabGlass, Vineland, NJ, USA, Cat. no.: BMS-005B).
- Bruker Avance III 600 MHz spectrometer with a cryoprobe (Bruker, Billerica, MA, USA).
- Abbe Mark III refractometer (Reichert, Buffalo, NY, USA).
- ZEN0040 cuvette.

## 3. Procedure

### 3.1. Plasmid Transformation and Expression Test in E. coli

- Under sterile conditions, saposin C plasmids are transformed into *E. coli* BL21 (DE3) cells using the heat shock method (*vide infra*). pET-30b vector encodes for kanamycin resistance. Thus, all steps involving LB or minimal media need a final kanamycin concentration of 50 µg/mL.
- Plasmid Transformation:
  - Add 1 µL of 100 ng DNA to cells and incubate on ice for 30 min.
  - Place in 42 °C water bath for 45 s.
  - Transfer to ice for 2 min.
  - Add 250 µL of provided S.O.C. medium and place in shaker for 1 h at 37 °C, 225 rpm.
  - Plate 100 µL of cell culture on LB agar plate and allow colonies to grow overnight at 37 °C.
  - The presence of single colonies the next morning indicates that an expression test can be performed in the evening.
- Expression test
  - Two 50 mL falcon tubes are filled with 5 mL of LB. A single colony is transferred to each tube and placed in a shaker incubator overnight at 37 °C, 225 rpm.
  - Check the optical density of culture at 600 nm (OD600) the next morning. If the OD600 is > 1.5, retain 1 mL of culture and dilute with 4 mL of fresh media.
  - The OD600 after dilution should be from 0.8 to 1. In this case, perform the following steps: 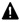 **CRITICAL STEP** Create a glycerol stock by taking 850 µL of culture and add 150 µL 100% glycerol, mix gently and store at −80 °C.
  - Extract a 60 µL aliquot of culture for a pre-IPTG SDS-PAGE sample.
  - Induce with 1 mM IPTG for 4 h at 37 °C, 225 rpm.
  - Once the 4 h induction period is complete, check the OD600 and scale the volume of culture for a pre-IPTG OD to achieve roughly the same number of cells when centrifuged. This serves as a post-IPTG SDS-PAGE sample.
  - To prepare the SDS-PAGE samples, centrifuge the bacterial culture and remove supernatant. There should be a small pellet at the bottom of the tube. Add 40 µL of 1x SDS dye and resuspend. Heat samples for 5 min at 95 °C and load onto gel.
  - If SDS-PAGE gel shows positive expression, the glycerol stocks created during this process will be used to provide a stater culture for a larger growth.

### 3.2. Protein Expression in Minimal Media

For NMR studies, it is advised to grow 4 L or more of bacterial culture due to low expression levels.

- Prepare each 1 L of minimal media in a 6 L flask to provide 5(x) more air for proper aeration of bacterial culture.
- For each 1 L of media transfer 250 mL into 500 mL flask and add 150 µL of glycerol stock and grow overnight at 37 °C at 225 rpm.
- Next morning check culture optical density at 600 nm (OD600) and if culture has an absorbance reading above 1.5, dilute starter culture by pouring the 250 mL into original 1 L in the 6 L flask.
- Check OD600 after diluting and once an OD600 of 0.8 to 1 is reached then induce with 1 mM IPTG.
- Induce culture for 4 h at 37 °C and centrifuge cells at 8000 rpm for 30 min. Discard the media and keep the cells. 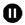 **PAUSE STEP:** The cells can be stored frozen below −20 °C.

### 3.3. Protein Purification

Prepare the reagents for protein resuspension and chromatography as indicated in Section 2.1.4.

- PMSF solution (1 µL of PMSF per 1 mL of resuspension solution) is added every 30 min.
- Remove cells from centrifuge bottles and place in beaker. Wash centrifuge bottle twice with 20 mL of nickel column buffer A (40 mL total) and transfer wash to beaker with cells.
- Stir cells with buffer for 30 min at 4 °C to break clumps prior to sonication.
- Sonicate resuspension using a Sonifier with the following settings: 70% amplitude, pulsing on and off for 15 and 45 s, respectively (48 min total).
- Keep solution on ice during sonication as the process can heat the cell lysate.
- Transfer to an ultracentrifuge and centrifuge for 30 min at 35,000 rpm at 4 °C.
- Filter supernatant through a 0.45 µm PES filter and take a sample for SDS-PAGE.
- Lightly scrape pellet with 10 mL pipette tip (smaller than the tip of a pencil) and rub pellet onto the walls of a 1.5 mL microcentrifuge tube and wash pellet twice with 200 µL of water, centrifuging in between to remove wash. Resuspend pellet with 40 mL of 1x SDS-PAGE dye.
- If SDS-PAGE gel revels a significant fraction of the protein remaining in the pellet, resuspend pellet with 20 mL of nickel column buffer A, continue stirring for 30 min and centrifuge again.
- Nickel chromatography is performed using a HPLC with a 5 mL prepacked column. 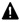 CRITICAL STEP: The recommended column flow rate is 1 mL/min. Thus, 5 min is equivalent to 1 column volume (CV).
- Purge both nickel column buffers A and B into the system and equilibrate the column by washing with water for 20 min (4 CV), then directly switching to buffer A for an additional 20 min.
- An instrument method is created using the time steps shown in Table 1 for the injection and elution of the protein always at a flow rate of 1 mL/min.
- The instrument method shown in Table 1 accounts for a 5 mL injection of the filtered lysate. For injection volumes larger than 5 mL of protein lysate, the first step needs to be extended to account for the additional volume of flowthrough.
- Samples are collected from the elution peaks based on optical absorbance values during the gradient and tested by SDS-PAGE to determine protein purity. The purest fractions are pooled together and are subjected to dialysis to remove excess salt in preparation for thrombin cleavage.
- Protein concentration after dialysis is determined by UV-Vis for constructs containing PUMA peptide (due to the presence of Trp in the amino acid sequence).
- Protein is subjected to thrombin cleavage overnight by adding 2 µL thrombin/1 mg of protein.
- Thrombin cleavage is assessed the next morning by SDS-PAGE. If >80% of cleavage is determined, sapC protein construct is separated from thrombin by HPLC using the C4 reverse phase column. The instrument method uses the same time points as the nickel column, but with reverse phase buffers A and B running at a flow rate of 5 mL/min.
- Protein purity is determined using SDS-PAGE and the purest fractions are pooled and frozen using liquid nitrogen then subjected to lyophilization for two days to ensure complete solvent removal.

### 3.4. Liposome Preparation

- Dry lipids in chloroform under vacuum and transfer to the lyophilizer overnight. Subsequently, hydrate in water for 1 h to a final concentration of 2 mM lipid.
- Vortex the solution for 10 min by securing the microcentrifuge tube to the vortexer with tape to create multilamellar vesicles (MLV).
- Bath-sonicate the MLVs for 20 min with ice.
- Liposome size and dispersity were checked by dynamic light scattering.

### 3.5. Conversion of Lipid Concentration to Liposome Concentration

- The conversion of lipid concentration in the mM range results in liposome concentration in the nM range.
- The liposome size determined by Dynamic Light Scattering (DLS) can be considered as the diameter of the outer membrane size and the inner membrane can be calculated as indicated in Equation (1), with the assumption that the lipid bilayer is 4 nm.
  r_i_ (nm) = r_o_ (nm) − 4 nm (1)
  where r_i_ is the radius of inner membrane, and r_o_ is the radius of outer membrane both expressed in nm.
- Determine the surface area (SA) of the inner and outer membrane using Equation (2).
  SA = 4πr^2^ (2)
- Divide the surface area of the membrane by the surface area of the head group (0.64 nm^2^) to determine the total number of lipid molecules in the inner and outer membrane. Add the values of the inner and outer membrane to obtain the total lipid molecules per liposome.
- Convert concentration of lipids to molecules of lipids using Avogadro’s number. Using the value determined in the previous step, convert lipid molecules to liposome assemblies.
- Convert the number of liposome assemblies to molar concentration using Avogadro’s number.

### 3.6. NMR Sample Preparation for Liposome Titration

- For all liposome titration experiments, 100 µM protein concentration is used with increasing amounts of liposomes. To achieve the same concentration of protein throughout the titration, a stock of 500 µM of protein is prepared and diluted for each individual sample. 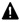 **CRITICAL STEP**: For NMR experiments a final concentration of 10% D_2_O is needed for frequency locking; therefore, our stock of 500 µM protein is prepared with 50% D_2_O to ensure that all samples have the proper concentration of both protein and D_2_O.
- Prepare protein stocks in HPLC-grade water at pH 6.0. Buffer is avoided to allow pH adjustments.
- Prepare each sample with a volume of 400 µL to compensate for small volume decrease due to pH adjustments.
- Table 2 lists values of protein and lipid concentration and volume used in NMR titration experiments.
- Adjust each sample to pH 6 after mixing.
- Add 300 µL into a Shigemi tube and insert the plunger avoiding air bubbles at the interface between the sample and glass. Secure plunger and tube with parafilm.

### 3.7. NMR Experiment Set Up for Liposome Titration

NMR experiments using cryogenic probes typically require millimolar or sub-millimolar protein concentrations. In addition, several experiments (approximately 8–10) are performed for NMR titrations. Thus, a significant amount of protein material is necessary at these concentrations, which can turn costly and time–consuming to produce. We recommend decreasing the protein concentration to micromolar values and use fast acquisition techniques [18] to avoid lengthy experiments that could be detrimental for unstable proteins.

- Acquire two-dimensional spectra at 298 K using fast-acquisition techniques: i.e., [^1^H-^15^N] SOFAST-HMQC [18].
- Calibrate the ^1^H pulse for each sample to estimate the power level of the pertinent 120° and 180° shape pluses (for convenience p15 and p20, respectively) using the calib-shape option from TopSpin 3.6.4 software.
  - Parameters for p15
    ○ Shape Pc9_4_120.1000
    ○ Length 3000 μs
    ○ Flip angle: 120°
  - Parameters for p20
    ○ ReBurp is the shape pulse for p20
    ○ Length 2000 µs
    ○ Flip angle: 180°
- Acquire each experiment with the same number of scans, dummy scans and receiver gain to ensure the signal to noise ratio is comparable between experiments during the titration. 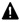 **CRITICAL STEP**: These experiments should be performed in duplicate for error determination.

### 3.8. NMR Experiment Set Up for Protein-Liposome pH Dependent Studies

- Use 1:1 protein:lipid molar ratio to determine the effect of pH dependency for liposome binding.
- Prepare stock solutions of saposin C and PC:PS at pH 6.8.
- Prepare a 4:1 molar ratio of PC:PS liposomes using the method described in Section 3.4.
- Mix protein and liposome solution at pH 6.8 as starting point of the pH titration.
- Adjust pH using sub-microliter volumes of acid and base solutions (HCl and NaOH).
- Acquire [^1^H-^15^N]-2D experiments at various pH values to observe signal decrease upon liposome binding.
- The pKa associated with protein–liposome binding is obtained by fitting the experimental NMR data to the Henderson-Hasselbalch equation (Equation (3)).
  (3) $$\mathrm{pH}={\mathrm{pK}}_{a}+\log_{10}\frac{\left[A^{-}\right]}{\left[\mathrm{HA}\right]}$$

### 3.9. NMR Data Processing

- Use Bruker Topsin software for data processing.
- Extract the 1D projection of the first serial file for each 2D experiment.
- Apply phase and baseline correction to the 1D spectrum.
- Use the first experiment of the titration set (100 μM sapC, no liposomes) as a reference for all subsequent experiments conducted with the same stock solution of protein.
- By integration, calculate the area within the NMR signals from 9.3 ppm to 6.6 ppm.
- Normalize the area with respect to the first sample (no liposomes).
- Determine the integral of all subsequent samples by selecting the option “Use Lastscale as Calibration” from the Topspin software for the same range.
- Each normalized integral value indicates the amount of protein remaining in solution; therefore, the relative amount of protein bound to liposomes is calculated with Equation (4).
  Relative amount of protein bound to liposomes = 1 − integral,(4)
- To determine the K_Dapp_ for the binding between protein and liposome, apply the Hill equation (Equation (5)).
  (5) $$\left[\theta \right]=\frac{{\left[L_{t}\right]}^{n}}{{\left[L_{t}\right]}^{n}+K_{D}}$$

### 3.10. Liposome Fusion

- Liposome fusion was determined using dynamic light scattering (DLS).
- Set up parameters for experiment: Refractive index of both protein and liposomes is 1.33, and absorbance at 632.8 nm is 0.05.
- Take measurements using the “size” setting with 3 readings per measurement and equilibration time of 120 s.
- Select the appropriate cuvette. The ZEN0040 cuvette for 160 µL of sample volume is appropriate.
- Prepare protein samples for a final protein concentration of 100 µM upon dilution with liposomes. PS liposomes were prepared as described above in Section 3.4
- Filter protein sample through 0.05 µm PES filter and check the initial size and polydispersity prior to liposome addition.
- SapC and sapC-PUMA constructs were prepared at pH 4.3 and 6.0, respectively.
- Filter liposome sample prior to protein addition to ensure monodispersity. Initial liposome size is determined for time 0.
- SapC constructs and liposomes were mixed to achieve a final concentration of 100 µM
- SapC and sapC-PUMA constructs were mixed at pH 4.3 and 6.0, respectively. Subsequently, sapC-PUMA solution was adjusted to 5.3.
- The protein-liposome solution was spun down to pellet any precipitation.
- The supernatant is then placed into cuvette and tested for liposome fusion. Liposome fusion should immediately begin post centrifugation. For kinetic experiments measurements were taken every 20 min for 200 min.

## 4. Expected Results

Saposin C constructs bind to liposomes at acidic or mildly acidic pH. There are three main factors affecting the binding process: solution pH, lipid concentration and type of lipid used to produce liposomes. Binding studies can be performed *via* titration of the protein with different concentrations of liposomes at a constant pH value or by changing the pH at a constant lipid concentration. In general, it is expected that pH titrations at constant liposome concentration will result in more sapC binding upon further acidification [2]. Analogously, enhanced protein–liposome binding is expected at constant pH and increased liposome concentration [6].

**Figure 3:**
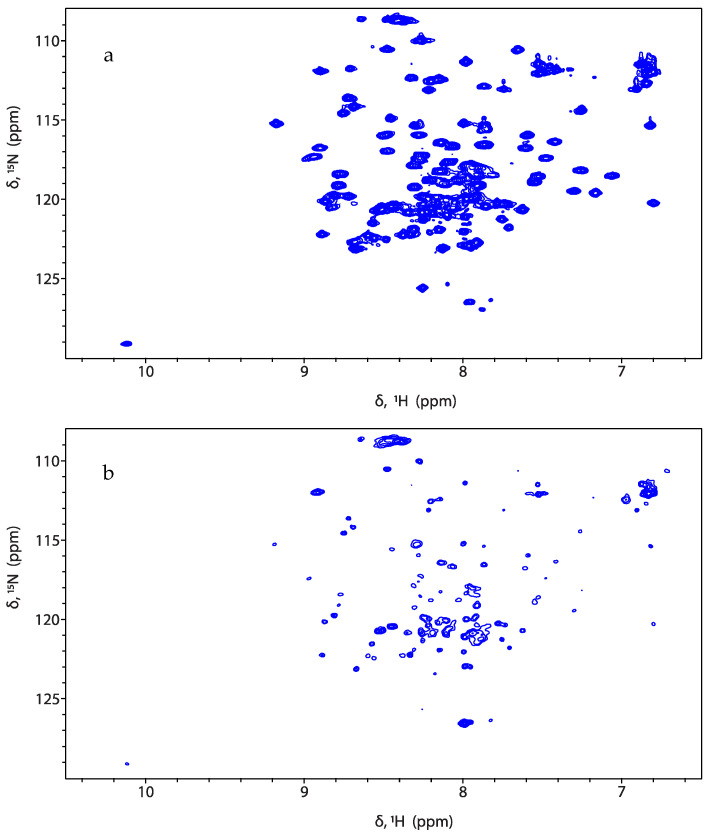
SapC-PUMA binding to liposomes results in decreased signal intensity. 2D [^1^H-^15^N]-SOFAST-HMQC [18] spectra of sapC-PUMA at pH 6.0 in the absence of liposomes (**a**) and at 1:5 molar ratio of sapC-PUMA:PS lipids (**b**).

**Figure 4:**
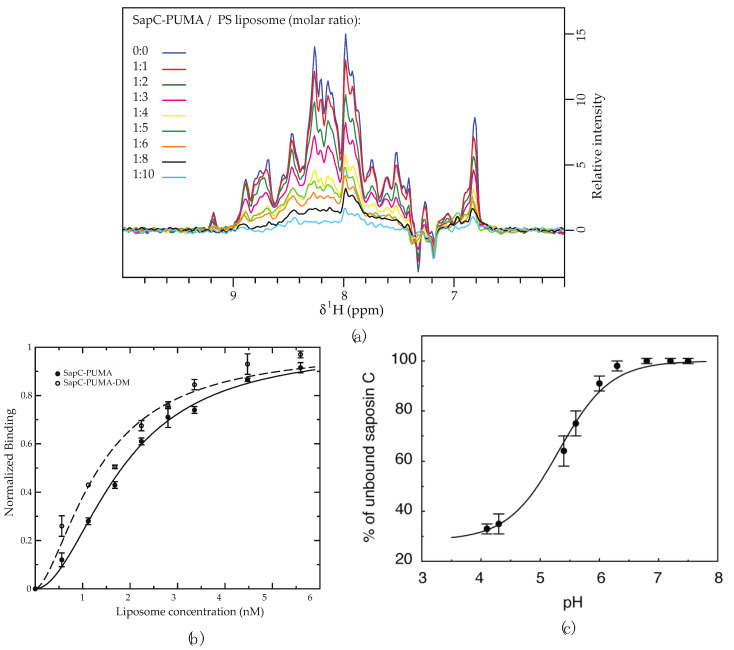
The binding of sapC constructs to liposomes results in NMR signal intensity decrease. (**a**) Signal intensity of sapC-PUMA amide ^1^H of 1D projections from 2D [^1^H-^15^N]-SOFAST-HMQC [18] decreases with increasing lipid concentration. In the absence of liposomes (dark blue) the signal intensity is normalized to 1. (**b**) The binding affinity of sapC-PUMA constructs to liposomes can be determined by fitting to the Hill equation. Experiments are conducted in duplicate. Bars indicate experimental errors. (**c**) SapC binding to PC:PS liposomes increases under acidic conditions [2]. (**a**,**b**): Reprinted from Pharmaceutics, 13 (2021) 583 (**c**): Reprinted from Biochemistry, 42 (2003) 14,729−14,740. Published 2003 American Chemical Society.

Both titration experiments leverage the significant change in particle size upon binding to liposomes, which slows the effective tumbling rate of the ^15^N-labeled protein constructs. Very slow tumbling rates of the proteoliposomes broaden NMR protein signals turning them undistinguishable from the baseline noise level. Under these conditions, only NMR signals from the free protein (unbound) will be observed. This effect is depicted in Figure 3 and Figure 4a, where the NMR signal intensity of the protein sapC-PUMA decreases with the increase in liposome concentration. The decrease in NMR signal intensity indicates protein binding to liposomes (invisible to NMR), thus depleting the solution of free protein (visible to NMR). The change in NMR signal intensity with liposome concentration can be fitted to the Hill equation to determine apparent dissociation constants (K_Dapp_) (Figure 4b). The K_Dapp_ found for sapC-PUMA is 3 nM with a Hill coefficient of ~ 2, which indicates the number of adsorbed molecules per binding site in agreement with the dimerization mechanism.

Importantly, this method allows to identify changes in the affinity of protein membrane binding. In fact, the protein sapC-PUMA-DM, with two mutations in the sapC domain of the chimera designed to enhance liposome binding, shows a decreased value of the K_Dapp_ (1.6 nM) [6]. The two mutations replace Asp 52 and Glu 64 in sapC for Arg to reduce the overall negative charge of sapC’s electrostatic surface and promote interactions with the negatively charged heads of the phospholipids [2,6].

In addition, NMR titration experiments can also be used to identify the optimal pH for sapC binding to liposomes, resulting in ~70% bound protein at pH 4.2 in the presence of PC:PS liposomes [2] (Figure 4c). The fitting of the NMR titration data to the Henderson–Hasselbalch equation allows us to determine an apparent binding pKa of 5.3. The slight deviation from the sigmoidal behavior is likely due to the simultaneous titration of several Glu and Asp side chains in sapC. In fact, the electrostatic surface of sapC is highly negatively charged [2]; the charge is partially neutralized upon acidification, thus facilitating interactions with the hydrophobic chains of the phospholipids.

For certain biotechnological or medical applications of sapC proteoliposomes, it might be necessary to work at mildly acidic conditions. It has been shown that sapC binds preferably to PS lipids as compared to PC or PC/PS mixtures [19]. For this reason, we use PS-only liposomes in the titration experiments to determine the apparent dissociation constant for sapC-PUMA constructs as PS optimizes binding at less acidic conditions (pH 6.0) [6]. We found that the Hill equation is appropriate for determining the K_Dapp_ for liposome binding as it accounts for cooperativity upon binding [20]. SapC and many other saposin-like proteins are known to dimerize [21,22,23,24], suggesting that the Hill coefficient is necessary to account for multiple proteins binding to a single site.

Some SAPLIPs, such as SP-B, have transient interactions with lipid membranes to facilitate lipid transfer [12]. These transient interactions will likely result in line broadening of NMR signals. Thus, the method as describe here will not be applicable; however, an analogous method that monitors signal line broadening can be devised for such interactions.

**Figure 5:**
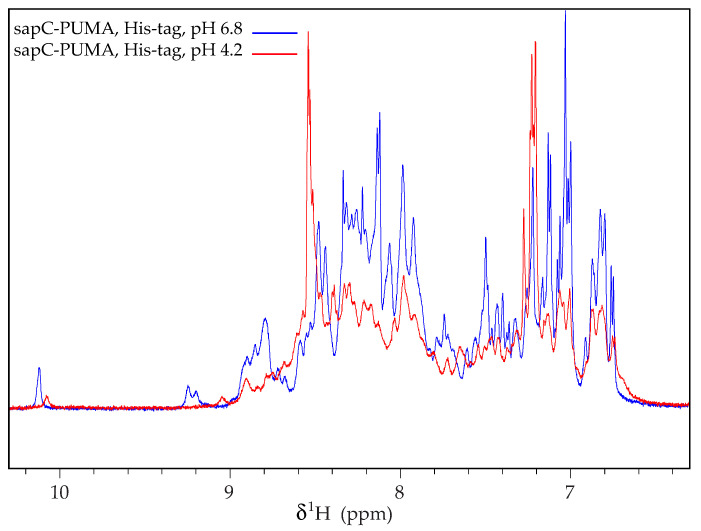
SapC-PUMA aggregates at acidic pH when the His-tag is not removed. 1D ^1^H-NMR spectrum of sapC-PUMA at 6.8 and 4.2 in the presence of His-tag. Reprinted from Pharmaceutics, 13 (2021) 583.

Importantly, the protein purification protocol describes the role of the protease thrombin to remove the His-tag used for purification. This step is necessary because our NMR data indicate that sapC constructs aggregate in the presence of the His-tag at acidic pH (Figure 5). NMR signals become broader for sapC-PUMA when subjected to pH changes from 6.8 to 4.2 if the His-tag is not removed (Figure 5). In contrast, this behavior is not observed for sapC or sapC-PUMA constructs without His-tag after pH adjustment from neutral to acidic conditions [2,6].

In principle, the titrations between SAPLIPs and liposomes can be performed in unlabeled proteins using ^1^H 1D-NMR. However, we do not recommend this option because (1) it might be difficult to discern liposome binding from NMR signal broadening, should the protein aggregate; (2) if the presence of liposomes promotes conformational changes in the free protein due to transient protein-lipid interactions, this information could be easily lost in 1D NMR; (3) 1D NMR does not allow to fully discern that the protein conserves the original fold at the different pH values used in the titrations experiments. The use of fast acquisition techniques [18] allows to test protein concentrations in the low micromolar range instead of millimolar concentrations typically used in NMR, which might have important budgetary implications. For the same reason, if liposome-binding proteins tend to self-associate, fast acquisition techniques will facilitate the binding studies as they have been extensively used to monitor protein interactions of self-assembling proteins [25,26,27,28,29].

Finally, it is known that sapC and SAPLIPs can fuse liposomes [5,6,22]. This is another relevant characteristic of the function of SAPLIPs. Fusogenic properties can be leveraged for important biomedical applications involving drug delivery due to the potential fusion of proteoliposomes and cell plasma membranes. DLS is an ideal technique for liposome fusion studies, although fusion can also be assessed with size exclusion chromatography and transmission electron microscopy [19]. In contrast to the latter two, DLS allows to monitor fusion in real time, thus providing an estimate of the time required for fusion to occur, the final polydispersity of the liposome solution and liposome largest size achieved.

**Figure 6:**
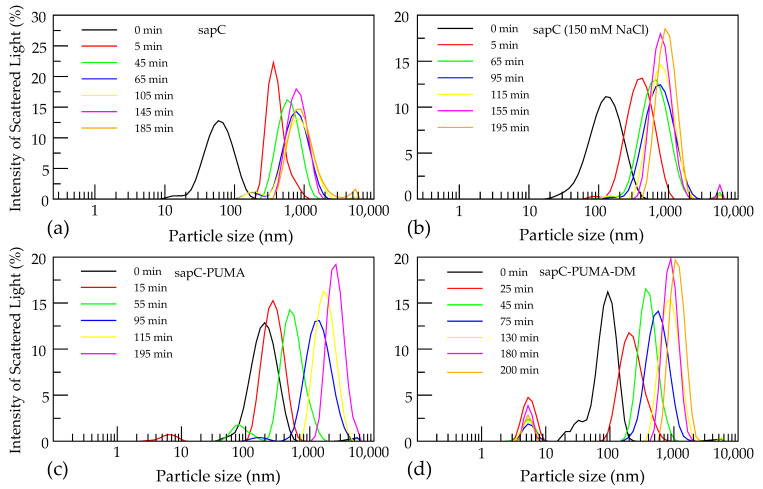
SapC constructs induce liposome fusion. Initial liposome size is shown in black at ~100–200 nm. Peaks appearing below 10 nm correspond to unbound protein. The following conditions were tested: sapC in water pH 4.3 (**a**), sapC with 150 mM NaCl pH 4.3 (**b**), sapC-PUMA in water pH 5.3 (**c**), and sapC-PUMA-DM in water pH 5.3 (**d**). Reprinted from Pharmaceutics, 13 (2021) 583.

Prior to fusion, the initial conditions of the liposome sample should be checked, aiming for monodisperse solutions of liposomes with diameters in the 100–200 nm range. Upon addition of sapC constructs, liposome fusion takes place with the most activity occurring in the first five minutes (Figure 6). In our experiments, liposomes show a nine-fold increase in size over the course of 200 min [6]. No additional changes in liposome size are observed at longer times.

## Figures and Tables

**Table 1:** HPLC nickel-affinity purification method.

Time (min)	Buffer	Step
0–20	Buffer A	Flow through passes through column
20–50	Gradient A to B	Protein is eluted
50–55	Buffer B	Column is cleaned
55–60	Gradient B to A	
60–75	Buffer A	Column is re-equilibrated

**Table 2:** Concentration and volume of protein and lipid stocks used in NMR titration experiments.

Protein:Lipid Ratio	Volume of 500 µM SapC-PUMA (µL)	Volume of 2 mM Lipids (µL)	Water (µL)
1:0	80	0	320
1:1	80	20	300
1:2	80	40	280
1:3	80	60	260
1:4	80	80	240
1:5	80	100	220
1:6	80	120	200
1:8	80	160	160
1:10	80	200	120

## Data Availability

Data supporting reported results can be found in Pharmaceutics, 13 (2021) 583.
